# The Metabolomics of Childhood Atopic Diseases: A Comprehensive Pathway-Specific Review

**DOI:** 10.3390/metabo10120511

**Published:** 2020-12-16

**Authors:** Mette S. Schjødt, Gözde Gürdeniz, Bo Chawes

**Affiliations:** 1 COPSAC, Copenhagen Prospective Studies on Asthma in Childhood, Herlev and Gentofte Hospital, University of Copenhagen, 2820 Copenhagen, Denmark; meschj@rm.dk (M.S.S.); gozde.gurdeniz@dbac.dk (G.G.); 2 Department of Pediatric and Adolescent Medicine, Herlev and Gentofte Hospital, University of Copenhagen, 2730 Copenhagen, Denmark

**Keywords:** asthma, atopy, metabolomics, children, short-chain fatty acids, polyunsaturated fatty acids, bile acids, tryptophan, tyrosine, lipids

## Abstract

Asthma, allergic rhinitis, food allergy, and atopic dermatitis are common childhood diseases with several different underlying mechanisms, i.e., endotypes of disease. Metabolomics has the potential to identify disease endotypes, which could beneficially promote personalized prevention and treatment. Here, we summarize the findings from metabolomics studies of children with atopic diseases focusing on tyrosine and tryptophan metabolism, lipids (particularly, sphingolipids), polyunsaturated fatty acids, microbially derived metabolites (particularly, short-chain fatty acids), and bile acids. We included 25 studies: 23 examined asthma or wheezing, five examined allergy endpoints, and two focused on atopic dermatitis. Of the 25 studies, 20 reported findings in the pathways of interest with findings for asthma in all pathways and for allergy and atopic dermatitis in most pathways except tyrosine metabolism and short-chain fatty acids, respectively. Particularly, tyrosine, 3-hydroxyphenylacetic acid, N-acetyltyrosine, tryptophan, indolelactic acid, 5-hydroxyindoleacetic acid, p-Cresol sulfate, taurocholic acid, taurochenodeoxycholic acid, glycohyocholic acid, glycocholic acid, and docosapentaenoate n-6 were identified in at least two studies. This pathway-specific review provides a comprehensive overview of the existing evidence from metabolomics studies of childhood atopic diseases. The altered metabolic pathways uncover some of the underlying biochemical mechanisms leading to these common childhood disorders, which may become of potential value in clinical practice.

## 1. Introduction

Asthma, allergic rhinitis, food allergy, and atopic dermatitis (AD)—the atopic diseases—are common childhood diseases with prevalences that vary across geographical regions, age groups, and disease severity. On a global scale, the prevalence of asthma is approximately 14% in children [1], allergic rhinitis is diagnosed in up to 15% of children [2,3], food allergy in up to 8% of children [4], and AD in up to 20% of children [5]. These diseases, particularly asthma, are very heterogeneous with several underlying pathophysiological mechanisms, i.e., endotypes of disease [6,7], all of which may require different terms of prevention and treatment. Molecular endotypes of AD [8] and allergic rhinitis have also been described [9]. There has been an increased interest in asthma metabolomics in recent years, as it provides an insight into pathways leading to different endotypes of asthma. Fluctuations in metabolites represent an integrated pathophysiologic profile involving environmental exposures and genetics [10] and the interplay between those mediated by an epigenetic mechanism [11]. Thus, metabolomics profiling of various biofluids may differentiate between asthma endotypes and potentially help tailor asthma treatment for the individual child.

Metabolomics studies have shown that asthma in children and adults is associated with alteration of levels of particular metabolites and pathways, e.g., metabolism of the amino acids tyrosine and tryptophan; lipids, such as sphingolipids and phospholipids; polyunsaturated fatty acids (PUFAs); bile acids; and microbially derived metabolites from the gut, including short-chain fatty acids (SCFAs) [10,12]. Previous reviews have studied asthma-associated metabolites and pathways across biospecimens in both children and adults (i.e., serum, plasma, urine, exhaled breath condensate (EBC), and volatile organic compounds (VOCs) [10,13] and gut microbial-derived metabolites from stool samples) [12]. To date, no review has summarized the evidence from asthma metabolomics studies in children, focusing on the pathways previously mentioned or encompassed metabolome associations related to allergic rhinitis, food allergy, and AD in children. Therefore, we aimed to summarize results from metabolomics studies of atopic children focusing on the metabolism of tyrosine and tryptophan, lipids, PUFAs, SCFAs, and bile acids to investigate a potential role of metabolites in these pathways for discriminating between health and atopic diseases and for identifying different endotypes of atopic disease.

## 2. Results

### 2.1. Study Selection and Characteristics

**Figure 1:**
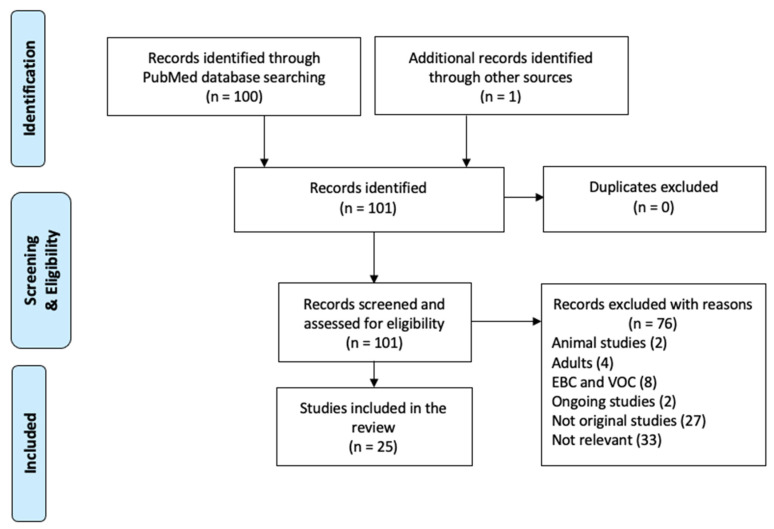
Flowchart of the study selection process.

A total of 100 records were identified through the database search, where 24 of these studies fulfilled the inclusion criteria. An additional study [14] was found by reviewing references (Figure 1). Thus, a total of 25 studies were included in this review.

Characteristics of the included studies are presented in the Supplementary Materials (Table S1). Asthma was examined in 19 studies [14,15,16,17,18,19,20,21,22,23,24,25,26,27,28,29,30,31], wheeze in six [14,19,32,33,34,35], AD in two [36,37], and allergy (allergic rhinitis or food allergy) in five studies [16,17,18,35,38]. The number of atopic children with either asthma, wheeze, allergy, or AD ranged from 20 [14,36] to 380 [27]. A total of 20 studies were case-control [15,16,17,18,19,21,23,24,25,26,27,29,31,32,33,34,35,36,37,38], four included cases but no controls [14,20,22,28], and one was a cohort study [30]. Six studies included children aged 6 to 18 years [15,20,25,26,27,28], 10 included children younger than six years [18,19,31,32,33,34,35,36,37,38], and eight studies included children in different age groups ranging from four weeks to 18 years [14,16,17,21,22,23,24,30]. One study included children aged 9 to 19 years [29]. Atopic conditions were primary physician diagnosed (*n* = 18) [14,15,17,18,19,20,21,22,23,24,25,27,28,30,32,36,37,38], based on parental report of a physician’s diagnosis (*n* = 2) [26,31], or either physician diagnosed or parental reported (*n* = 3) [16,29,35]. Parental-reported wheezing was used in two studies [33,34]. Distribution of examined biospecimens were urine (*n* = 12) [15,18,19,20,21,22,24,30,32,33,35,36], plasma (*n* = 5) [14,25,26,27,28], serum (*n* = 3) [16,29,37], stool (*n* = 2) [17,31], amniotic fluid (*n* = 1) [34], and two studies investigated samples from urine and either serum or plasma [23,38]. A total of 18 studies used MS methods [14,15,16,19,20,21,22,25,26,27,28,29,30,31,32,34,35,37] and seven used NMR [17,18,23,24,33,36,38], with 20 studies using untargeted metabolomics [15,16,17,18,19,20,21,23,25,26,27,28,30,31,32,33,34,35,36,38] and four a targeted method [14,22,24,29]. One study used both targeted and untargeted methods [37].

### 2.2. Pathway-Specific Study Results

All reported study findings are presented below according to the specific metabolic pathways of interest. The summary of results by biospecimens is presented in Table 1.

Metabolites within the pathways of interest identified as significant in more than one study are shown in Table 2, and implicated pathways of interest across studies are shown in Table 3.

**Figure 2:**
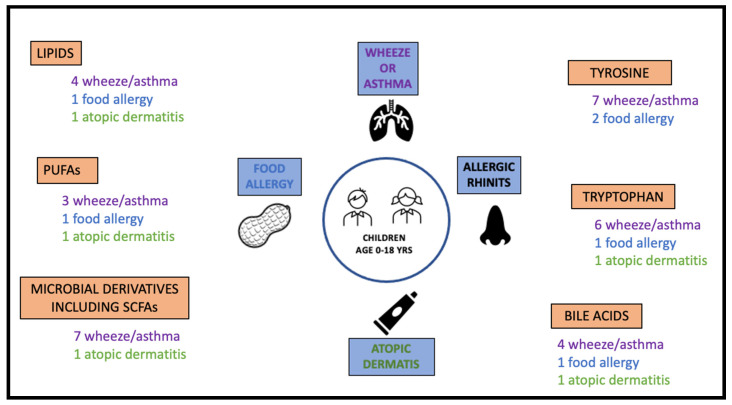
The number of studies showing significant associations between atopic diseases and metabolic pathways of interest.

**Figure 3:**
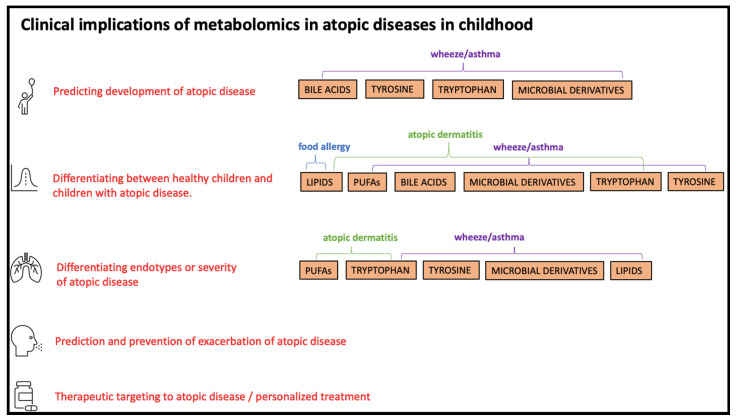
Clinical implications of metabolomics in atopic diseases related to pathways of interest in at least one of the included studies.

The number of studies showing significant associations between childhood atopic diseases and metabolic pathways of interest are illustrated in Figure 2. Clinical implications of metabolomics in atopic diseases in childhood related to pathways of interest are illustrated in Figure 3.

### 2.3. Tryptophan and Tyrosine Metabolism

#### 2.3.1. Asthma and Asthma Subtypes

The tryptophan and tyrosine metabolism and the metabolites involved in these pathways were associated with childhood asthma, distinctly in metabolomic studies using urine. Tao et al. [15] found both tryptophan and tyrosine to be upregulated in children with uncontrolled asthma compared to healthy controls (HCs) (*p* < 0.05), whilst only tyrosine metabolism was significant in a pathway enrichment analysis (*p* < 0.001). Moreover, the metabolite tyrosine could distinguish the severity of asthma between children with uncontrolled and controlled asthma (fold change, 1.542, *p* = 0.018). This result is in line with Saude et al. [24], who showed that tryptophan and tyrosine could differentiate stable asthma from unstable asthma in children. In addition, Saude et al. [24] found that tryptophan and tyrosine could separate children with stable asthma from healthy children, in contrast to Tao et al. [15], who reported no difference between children with controlled asthma and HCs. Additionally, the fecal level of tryptophan did not differ in children with asthma or allergic rhinitis, respectively, compared to HCs in a study by Chiu et al. [17].

Carraro et al. [19] found higher levels of five microbial tryptophan metabolites in children with “early-onset asthma” compared to children with “transient wheezing”, these metabolites being indole, glutaric acid, 5-hydroxy-1-tryptophan, indole-3-acetamide, and 3-indoleacetic acid. Contrary, children with “transient wheezing” had a higher level of indolelactic acid, which is a breakdown product of tryptophan metabolism and L-tyrosine (All *p* < 0.05). Hydroxyphenyllactic acid, a tyrosine metabolite, was elevated but not statistically significant in children with “early-onset asthma” (*p* = 0.058). However, Tao et al. [15] reported that the urinary level of hydroxyphenyllactic acid was increased in children with uncontrolled asthma compared to HCs, whereas 3-hydroxyphenylacetic acid was increased in HCs compared to asthmatics (*p* < 0.05).

Papamichael et al. [22] investigated the relationship between 34 urinary metabolites and lung function parameters (spirometry and peak flow) and fraction of exhaled nitric oxide (FeNO) in children with mild asthma. They reported a negative correlation between 4-hydroxyphenylacetic, which is involved in bacterial degradation of L-tyrosine to tyramine, and forced expired volume in the first second (FEV1) as well as forced vital capacity (FVC). Papamichael et al. [22] also reported a positive correlation between 5-hydroxyindoleacetic acid (5-HIAA) and the FEV1/FVC-ratio and a negative correlation between 5-HIAA and FeNO (*p* < 0.05). Tao et al. [15] reported that 5-HIAA could separate HC from children with uncontrolled and controlled asthma (*p* < 0.05).

Checkley et al. [29] found a lower level of shikimate-3-phosphate in serum of children with asthma compared to HCs (*p* = 0.001).

#### 2.3.2. Asthma Treatment

Quan-Jun et al. [23] demonstrated that combined treatment with inhaled corticosteroids (ICSs) and β2-agonists during an asthma exacerbation altered levels of 21 metabolites in urine and 22 metabolites in serum. 5-HIAA was increased in both urine and serum (*p* < 0.05).

Park et al. [20] examined urinary metabolites associated with ICS resistance in children with severe asthma. They found levels of two metabolites in tyrosine metabolism: 3,4-dihydroxy-L-phenylalanine and 3-methoxy-4-hydroxyphenyl(ethylene)glycol were both elevated in ICS-resistant compared to ICS-responding children (*p* = 0.05). Moreover, pathway analysis revealed that tyrosine metabolism was affected by corticosteroid resistance (*p* = 0.01).

#### 2.3.3. Wheeze/Asthma in Early Childhood

Other metabolites related to tryptophan and tyrosine have been shown in urine and amniotic fluid studies of asthmatic and wheezing children. The metabolite n-acetyltyrosine, a derivative of L-tyrosine, has also been investigated in two studies using urine samples by Chiu et al. [18,38], however, with inconsistent results. N-acetyltyrosine at age one year was significantly lower in children who developed asthma before turning 4 years old compared to HCs (*p* = 0.039) [18]. Nevertheless, N-acetyltyrosine and tyrosine levels were not significantly different between children with asthma at age 3 to 5 years and HCs [38]. Instead, both N-acetyltyrosine and tyrosine were significantly increased in children aged 3 to 5 years with vs. without food sensitization against milk or egg.

Turi et al. [33] examined infants with an acute viral respiratory infection and parentally reported wheezing during their first three years of life. In this study, urinary tyrosine and 4-hydroxyphenylacetate were associated with a reduced risk of first- and second-year wheezing (only tyrosine), although this finding was not significant after FDR corrections.

One study investigated metabolites in amniotic fluid samples. Carraro et al. [34] demonstrated an elevated level of 5-hydroxyindolepyruvate and 3-hydroxyphenylacetic in infants without compared to those with at least one parentally reported wheezing episode in their first year of life (*p* < 0.05). Additionally, pathway analysis showed that tryptophan and 2-phenylalanine metabolism were associated with wheezing in the first year of life (*p* = 0.009).

#### 2.3.4. Food Allergy and AD

Huang et al. [37] found higher levels of tryptophan and indolelactic acid in children with AD with elevated specific-IgE compared to those with normal IgE and HCs, respectively, whilst these metabolites did not differ among children with AD and normal IgE levels compared to HCs. Crestani et al. [16] identified that 81 metabolites were altered in children with food allergy compared to children with asthma. The metabolite 5-hydroxyindoleacetate in the tryptophan metabolism and the five metabolites: 3-(4-hydroxyphenyl)lactate, 3-methoxytyramine sulfate, 4-methoxyphenol sulfate, phenol glucuronide, and phenol sulfate in the tyrosine metabolism, were significantly different in children with asthma compared to children with food allergy. The authors concluded that children with a combination of asthma and food allergy capture a metabolomic signature concordant with food allergy alone rather than asthma [16].

### 2.4. Bile Acids

#### 2.4.1. Asthma/Wheeze

Several studies have found that bile acid metabolism is related to atopic diseases in children. Kelly et al. [26] demonstrated that asthma was associated with an elevated level of taurocholate (*p* < 0.05), a bile salt involved in fat emulsification, and downregulated biliverdin (*p* < 0.05), a bile pigment and product of heme catabolism. However, the association between asthma and bile acids were not replicated in another population of 411 children.

Crestani et al. [16] identified several bile acids in serum associated with atopic diseases. Three primary bile acids distinguished between children with asthma and non-atopic controls: taurochenodeoxycholate/taurochenodeoxycholic acid and glycocholate/glycocholic acid. Further, taurocholic acid and four secondary bile acids: glycohyocholate, glycoursodeoxycholate, and taurohyocholate, could significantly distinguish food allergy from asthma in children, even after adjustment for AD.

Other primary bile acids have also been associated with wheeze/asthma. Chawes et al. [30] investigated urinary biomarkers in four-week-old infants born to mothers with asthma from two Danish cohorts (COPSAC). Taurochenodeoxycholate-3-sulfate was higher in children who developed persistent wheeze before age three or asthma after age three (*p* < 0.05). Taurochenodeoxycholate-3-sulfate is a bile salt formed in the liver by conjugation of chenodeoxycholate with taurine. Carraro et al. [34] reported increased levels of the primary bile acids chenodeoxycholic acid 3-sulfate and the derivative of the secondary bile acid lithocholic acid, 3α-hydroxy-7,12-dioxo-5β-cholan-24-oic acid, in amniotic fluid in children without wheeze compared to children with wheeze during the first year of life. Glycocholic acid and the bile acid derivative 1,3,7,12-tetrahydroxycholan-24-oic-acid were elevated in the wheezing group. However, these results were not statistically significant.

At age three months, Arrieta et al. [35] showed that the urinary excretion of the sulfated bile acids glycolithocholate sulfate, glycocholenate sulfate, and glycohyocholate were significantly higher in children who developed atopy and wheeze at age one year compared to controls. In contrast, the excretion of tauroursodeoxycholate was decreased compared to controls (*p* < 0.001). In stool samples, Lee-Sarwar et al. [31] demonstrated that ursodeoxycholate sulfate, which is involved in the secondary bile acid metabolism, was inversely associated with asthma, but the result did not remain significant after correction for multiple testing.

#### 2.4.2. Asthma Treatment

McGeachie et al. [14] stratified children aged 1 to 18 years with wheeze or asthma into controlled or uncontrolled based on self-reported use of inhaled β2-agonist. They investigated 32 metabolites, including bile acids, fatty acids, and metabolites, in the sphingolipid pathway. None of the metabolites could differentiate between the groups based on the use of β2-agonist in the week preceding the blood sampling, and no pathway was associated with asthma control. However, Quan-Jun et al. [23] reported an increased taurine level in serum but not urine during acute asthma exacerbation after ICS and β2-agonist (*p* < 0.05). Taurine conjugates bile acids along with the amino acid glycine in the liver before the primary bile acids are secreted to the bile. Additionally, taurine and hypotaurine metabolism was one of the seven most influenced pathways that prompted the reprogramming of the combined treatment [23].

#### 2.4.3. Food Allergy and AD

Pathway analysis by Crestani et al. [16] revealed that one of the most dysregulated pathways associated with the presence of food allergy compared with asthma included secondary bile acid metabolism.

Huang et al. [37] showed that glycocholic acid and three primary conjugated bile acids: taurocholic acid, taurochenodeoxycholic acid, and glycochenodeoxycholic acid, were significantly decreased in serum of children with AD independently of high or normal IgE level compared to HC. On the other hand, children with AD and high IgE had elevated levels of two unconjugated bile acids: cholic acid and chenodeoxycholic acid, compared to HCs.

### 2.5. Microbial Derivatives

#### 2.5.1. Atopic Diseases and SCFAs

SCFAs, including butyrate, which is a by-product of the fermentation of dietary fiber by gut bacteria, have protective properties to inflammatory diseases in various animal models [39]. Chiu et al. [17] showed a reduced level of butyrate in stool samples from children with rhinitis (*p* = 0.054) and asthma (*p* = 0.009) compared to HCs. Moreover, a higher level of 4-hydroxybutyrate was reported in asthmatic children compared to HCs (*p* = 0.016) but not in children with rhinitis compared to HCs. In another study [38] based on urine samples, 3-hydroxyisobutyric acid could not distinguish children with asthma from HCs. In contrast, Tao et al. [15] reported significantly higher urinary 4-hydroxybutyric acid in HCs compared to uncontrolled and controlled asthma. Finally, 3-hydroxybutyric acid was increased in uncontrolled compared to controlled asthma, but HCs had an increased level compared to asthma cases (all *p* < 0.05).

Two other butyrates, 2-hydroxyisobutyric acid and 2-hydroxybutyrate, have been implicated in children with asthma or AD. In a subset analysis among infants with respiratory syncytial virus infection, Turi et al. [33] found that the urinary level of 2-hydroxyisobutyric acid was associated with a higher risk of developing wheeze at age three years, but the associations did not remain statistically significant after FDR correction. Saude et al. [24] showed that 2-hydroxyisobutyric acid in urine could distinguish stable asthma from HCs. Finally, Assfalg et al. [36] investigated urine samples from infants with AD, and their analysis indicated increased 2-hydroxyburyrate in infants with AD compared to HC.

Quan-Jun et al. [23] demonstrated that serum 3-hydroxybutyrate and 4-hydroxybutyrate were significantly increased in asthmatic children during acute exacerbations after ICS and *β*2-agonist treatment (*p* < 0.05).

In newborns, acetate is the principal fecal SCFA present [40]. The pattern of SCFAs is altered as the maturation of the gut microbiome takes place until the age of approximately 3 to 4 years [41]. Turi et al. [33] found an association between urinary acetate in healthy infants and the risk of subsequent second-year recurrent wheezing, but after adjusting for multiple testing, it was non-significant (*p* = 0.09).

#### 2.5.2. Asthma/Wheeze and p-Cresol Derivatives

p-Cresol derivatives have also been reported related to asthma in childhood. p-Cresol is a part of the putrefaction process by bacterial fermentation of protein in the large intestines and is excreted in feces and urine. p-Cresol and 4-hydroxyphenylacetic are microbial metabolites of tyrosine. Kelly et al. [26] reported that asthma was associated with reduced p-Cresol sulfate compared to HCs (*p* < 0.05) in fasting plasma samples, which was replicated in non-fasting plasma samples from an independent cohort (VDAART). Lee-Sarwar et al. [31] investigated stool samples in children aged three years from a subgroup of offspring from the VDAART cohort and found four metabolites involved in tyrosine metabolism were inversely associated with asthma: p-Cresol sulfate, o-sulfo-L-tyrosine, phenol sulfate, and cis-4-hydroxycyclohexylacetic acid, but only p-Cresol sulfate remained significant after correction for multiple testing (*p* = 0.001). Carraro et al. [19] found that the urinary level of p-Cresol was elevated in children with transient wheezing compared to early-onset asthma (*p* = 0.001). In amniotic fluid, Carraro et al. [34] reported that elevated p-Cresol glucuronide was associated with wheezing during the first year of life (*p* = 0.02).

### 2.6. PUFAs

PUFAs, including n-6-fatty acids, such as linoleic acid and arachidonic acid, are reported to be associated with asthma. Linoleic acid is an essential fatty acid used in the synthesis of arachidonic acid, a precursor in eicosanoid biosynthesis, e.g., prostaglandins, thromboxanes, and leukotrienes. These three eicosanoids are mediators in inflammation and immunity regulation. Contrary, n-3-fatty acids have an anti-inflammatory function.

#### 2.6.1. Asthma

Kelly et al. [27] found that plasma linoleic acid metabolism was significantly enriched and associated with airway hyperresponsiveness to methacholine and with the FEV1/FVC ratio at baseline and after bronchodilator (*p* < 0.05). Lee-Sarwar et al. [31] reported three n-6 and one n-3 PUFAs in stool were inversely associated with asthma: docosapentaenoate (n-6 DPA; 22:5n6), linoleate, adrenate, and docosapentaenoate (n-3 DPA; 22:5n3). However, only docosapentaenoate remained significant after correction for multiple testing (*p* = 0.0004). In a weighted gene correlation network analysis (WGCNA), 54 modules of highly correlated intestinal metabolites inversely associated with asthma were identified. One of these modules contained PUFAs, including docosapentaenoate, adrenate, octadecenedioate, docosapentaenoate, arachidonate, docosahexaenoate, eicosapentaenoate, dihomo-linolenate, and mead acid (*p* = 0.01). Additionally, Crestani et al. [16] found that serum arachidonate, dihomo-linolenate, and docosapentaenoate could discriminate children with asthma from children with food allergy after adjustment for AD (*p* ≤ 0.005).

Two studies investigated a targeted lipid panel, including eicosanoids, in children with asthma and AD. McGeachie et al. [14] found that no single metabolite or single pathway in plasma samples could discriminate between good and poor asthma control. Still, they demonstrated that arachidonic acid metabolism and linoleic acid metabolism possessed the highest pathway impact scores associated with asthma control status. Further, McGeachie et al. [14] incorporated gene expression and methylation data into their metabolomics analysis, showing that the most frequently observed metabolite was arachidonic acid, which appeared in 12 out of 17 pathways. In addition, PGE2 and sphingosine-1-phosphate were the second and third most represented metabolites among the pathways in the over-representation analysis.

#### 2.6.2. AD

Huang et al. [37] investigated targeted eicosanoids in serum of children with AD and showed different levels in children with AD with high IgE and normal IgE levels, respectively, compared to HC. Hydroxyl octadecadienoic acids (HODEs) and most of the investigated hydroxy eicosatetraenoic acids (HETEs) were significantly higher in the two AD groups compared to HCs. Children with AD and high IgE expressed significantly lower levels of 8, 9, 11, 12, 16, 19, 20-HETEs, and 9, 13-HODEs than those with normal IgE. Moreover, the levels of PGE2 and LTB4 were significantly higher in the AD groups. However, PGE2 and LTB4 could not discriminate AD groups with high IgE levels from normal IgE levels.

### 2.7. Lipids, Sphingolipids, and Ceramides

#### 2.7.1. Asthma

Several studies have linked glycerophospholipids, sphingolipids, and sphingolipid metabolism with asthma. In plasma samples, Kelly et al. [27] found that enrichment in the glycerophospholipid pathway was associated with airway hyperresponsiveness and the FEV1/FVC-ratio at baseline and after bronchodilator, whereas enrichment in the sphingolipid pathway was only associated with airway hyperresponsiveness. Lipid pathways were also associated with lung function in another study on plasma samples by Kelly et al. [25]. Metabolites were clustered by WGCNA into different modules, where the lipid module showed a trend of association with FEV1 in children with asthma (*p* = 0.057). Within this lipid module, baseline FEV1 correlated with glycerophospholipid-anchor biosynthesis (*p* = 0.034) and showed a trend of association with the sphingolipid metabolism (*p* = 0.061), glycerolipid metabolism (*p* = 0.077), and glycerophospholipid metabolism (*p* = 0.093). A total of 30 of the identified metabolites in the lipid module were also profiled in serum samples from a validation group of children (CAMP cohort), which showed a trend of association with FEV1 in a principal component analysis (*p* = 0.073).

In urine samples, Carraro et al. [19] found that phosphatidyl glycerol was elevated in children with early-onset asthma compared to transient wheezing (*p* = 0.049). Phosphatidyl glycerol is a glycerophospholipid found in pulmonary surfactant. Lee-Sarwar et al. [31] showed that the sphingolipid metabolite lactosyl-N-behenoyl-sphingosine was inversely associated with asthma, but the result was non-significant after correction for multiple testing. McGeachie et al. [14] did not find any association between the sphingolipid pathway (including 24 metabolites) and asthma control status. However, they showed that sphingosine-1-phosphate was involved in metabolite–gene association in asthma control and played a role in integrated omics analysis for predicting asthma control [14].

#### 2.7.2. Food Allergy and AD

Crestani et al. [16] showed that sphingolipid metabolism was affected by food allergy. Sphingolipid metabolism involves the conversion of ceramide into sphingomyelins and conjugated ceramides. The transformation of ceramides to sphingomyelin and acylceramide and glucosylceramide was decreased in children with food allergy compared to non-atopic controls and children with asthma (*p* < 0.05). Moreover, several lysophospholipids discriminated children with food allergy from asthmatic children and food allergy from non-atopic controls. Lower levels of phospholipids were associated with food allergy with or without concurrent asthma (all *p* < 0.05). The pathways strongly altered with the presence of food allergy included dihydrosphingomyelins, lactosylceramides, sphingomyelins, and hexosylceramides, among others.

In serum samples, Huang et al. [37] demonstrated significantly altered levels of three sphingomyelins in children with AD with elevated IgE compared to HCs.

## 3. Discussion

This pathway-specific review of tyrosine and tryptophan metabolism, lipids, PUFAs, gut microbial metabolites, and bile acids identified 25 studies that presented information on metabolomics profiling in children with asthma, allergy, or AD. The included studies varied considerably concerning age, biospecimens, technical approach, and diagnostic procedure. A total of 20 of the 25 studies reported significant metabolites or enriched pathways associated with either asthma, allergy, or AD in the prespecified pathways.

### 3.1. Tryptophan Metabolism

Tryptophan metabolites were significantly associated with atopic diseases in children. Three studies showed that metabolites within the tryptophan metabolism in urine [15,19,24] and serum [29] distinguished asthma from HCs [15,24,29] and early-onset asthma from transient wheezing [19]. The metabolite 5-hydroxyindolepyruvate in amniotic fluid could distinguish infants with and without wheezing episodes in the first year of life [34]. Moreover, tryptophan metabolites in serum could discriminate children with asthma from children with food allergy [16] and children with AD from HCs [37]. Two tryptophan metabolites, indolelactic acid and 5-HIAA, were identified as significant across studies, where the latter was positively correlated with the FEV1/FVC ratio and negatively correlated with FeNO [22], and increased after combination treatment of inhaled budesonide and salbutamol during acute asthma exacerbations [23].

Other studies have also reported significantly increased tryptophan levels in atopic children and adults. In a non-metabolomic study of 205 children, tryptophan and its downstream metabolite kynurenine were significantly higher in asthmatic children, allergic children, and in children with AD compared to HCs [42]. The highest tryptophan levels were observed in children with asthma, and in line with Saude et al. [24], tryptophan levels could significantly discriminate between controlled and uncontrolled asthma [42]. Tryptophan and kynurenine in serum were also significantly higher in adults with allergic asthma and allergic rhinitis compared to HCs [43,44]. Additionally, tryptophan was associated with eosinophilic inflammation and asthma symptom scores during experimental rhinovirus infection, but there was no association between tryptophan catabolism in the airways and exacerbation parameters [43]. However, other studies showed no difference in tryptophan levels in fasting or non-fasting plasma samples between asthmatics and HCs [43,45,46].

Although the role of tryptophan metabolism in asthma pathogenesis is still not fully understood, intermediates of tryptophan metabolism, produced via the kynurenine pathway and microbial catabolism, are important mediators of immune responses [47]. In murine models, tryptophan and its catabolites, including kynurenine, have been reported to play an anti-inflammatory role [48], which is in line with another study in which mice were fed with D-tryptophan before experimental asthma induction [49]. The study showed increased numbers of lung and gut regulatory T cells, decreased lung Th2 responses, and an ameliorated allergic airway inflammation and hyperresponsiveness [49].

A higher tryptophan level in subjects with atopy is suggested to result from lower IDO-1 (indoleamine 2,3-dioxygenase-1) activity, possibly because of the suppression of IDO-1 enzyme activity [50,51]. The enzyme IDO-1 is expressed in antigen-presenting cells and other cells resident in lymph nodes and inflammatory tissue [12] and catalyzes the rate-limiting step in tryptophan degradation in the kynurenine pathway and therefore reflects the kynurenine:tryptophan ratio. IDO-1 is induced by IFN-γ, which is responsible for the strongest Th1-mediated immune response against proinflammatory stimuli [51]. In children, IDO-1 enzyme activity was lower in patients with allergic rhinitis than asthmatic and in comparison to children with AD who exhibited the highest IDO-1 activity [42]. Furthermore, IDO-1 activity was higher in children with acute asthma and AD compared to children without exacerbations [42]. In adults, IDO-1 activity was lower in allergic asthmatic adults compared to HCs [43]. Lee-Sarwar et al. [31] highlights the anti-inflammatory and tolerogenic properties of IDO activity and kynurenine metabolites, including promoting T regulatory cells and reducing T cell inflammation due to a reduction in tryptophan availability.

In conclusion, studies demonstrate that tryptophan metabolism plays an important role in developing childhood atopic diseases, possibly affecting early-life immune homeostasis. More studies are needed to explore the role of tryptophan metabolism in asthma pathogenesis and the potential of therapeutics targeting this pathway in children.

### 3.2. Tyrosine Metabolism

The tyrosine pathway was altered in asthmatic children [15] and affected by ICS resistance [20]. In urine [15,18,19,24] and serum [29] samples, metabolites within the tyrosine metabolism differentiated asthma and asthma subtypes from HCs [15,24,29] and transient wheezing from early-onset asthma [19]. The metabolite 3-hydroxyphenylacetic in amniotic fluid could distinguish infants with and without wheezing episodes in the first year of life [34]. Moreover, urinary N-acetyltyrosine was associated with subsequent development of asthma [18]. The microbial metabolite p-Cresol and its derivatives produced from tyrosine were significantly associated with wheeze/asthma across biospecimens [19,26,31,34].

The evidence linking tyrosine metabolism and the pathogenesis of atopic diseases is limited. Some studies have suggested treating asthma with tyrosine kinase inhibitors [52,53,54,55] because the tyrosine kinase cascades may play a role in the pathogenesis of allergic airway inflammation [53]. Other studies have reported the derivate of L-tyrosine, bromotyrosine, to be associated with asthma, which is plausible as bromotyrosine results from eosinophil activation via eosinophil peroxidase during inflammatory responses [56]. Bromotyrosine may be a biomarker for monitoring asthma control and predicting exacerbations [57,58,59].

Plasma tyrosine levels were significantly lower in children with mild asthma compared to HCs in a non-metabolomic study by Sackesen et al. [45]. The authors suggested that oxidant injury of amino acids may be related to oxidative stress in asthma. Free amino acids are highly susceptible to oxidative stress by reactive oxygen species and modification of tyrosine (among other amino acids), resulting in oxidation products [45]. However, less is known about the antioxidant defense systems and the relationship between the oxidation products of amino acid residues and asthma [45].

In conclusion, further studies are needed to investigate the link between tyrosine metabolism and asthma and explore the potential of tyrosine-related therapeutics, including tyrosine kinase inhibitors and targeted antioxidant treatment.

### 3.3. Bile Acids

Metabolites within the primary and secondary bile acid metabolism were associated with atopic diseases in children. Taurocholic acid, glycocholic acid, glycohyocholic acid, and taurochenodeoxycholic acid were associated with wheeze [35], asthma [16,26,30], food allergy [16], or AD [37] in many studies.

One of the most dysregulated pathways associated with food allergy compared with asthma included secondary bile acids [16], which are formed by microbial actions in the colon. The intestinal microbiota alters bile acid composition or vice versa. The crosstalk between bile acids and intestinal microbiota has been shown to regulate lung immunity [30,60]. Moreover, bile acids have antimicrobial activity [12]. A protective effect of bile acids on allergic airway inflammation is reported in both in vitro and mouse studies [12]. Even though the link between bile acids and asthma is not well-studied in children, an untargeted metabolomics study in adults revealed a higher level of taurocholate in asthmatics compared to HCs [61]. Furthermore, asthmatics with high FeNO had significantly higher taurocholate levels compared to HCs [61]. NO modulates both bile acid metabolism and bile production [61], and therefore asthma severity, i.e., eosinophilic inflammation, may be related to bile acid metabolism. Sansbury et al. showed that transgenic mice with overexpressed endothelial NO synthase had increased levels of primary, secondary, and conjugated bile acids [62]. Severe eosinophilic asthma with high FeNO suggests a unique endotype related to changes in NO-associated taurine transport and bile acid metabolism [61]. In this regard, it is interesting that NO also is associated with the tryptophan-degrading enzyme IDO-1. Enhanced formation of NO has been reported in patients with asthma and allergic rhinitis [51], and NO suppresses the activity of IDO-1, which may explain the higher levels of tryptophan in asthmatics [51].

In conclusion, several bile acids were associated with asthma and other atopic diseases in children across the included studies. Thus, there is a potential to further explore the role of bile acid metabolism in asthmatic children.

### 3.4. Microbial Derivatives

Butyrate derivates of SCFA metabolism were significantly associated with asthma/wheeze [15,24,33,36] or AD [37] in five studies. SCFAs have been shown to play a critical role in several mechanisms that support putative protective effects on atopy and asthma [63]. They are reported to have several anti-inflammatory properties, including inducing regulatory T cells in mice [64,65,66], production of prostaglandin E2 [67], altering the function of dendritic cells [68], inhibition of histone deacetylase activity [69], limiting eosinophil trafficking and promotion [70], and survival of mucosal antibody production [71]. SCFAs are associated with a protective role against asthma and allergic diseases, including food allergy and allergic pulmonary inflammation models, directly or indirectly through changes in the intestinal microbiota [35,68,72,73]. SCFAs are produced through the fermentation of dietary fibers, and SCFAs may have therapeutic potential. In a particular study [68], mice were fed with a high-fiber diet, which affected their intestinal microbiota and increased the production of SCFAs. In turn, they were protected against allergic inflammation in the lung. In contrast, a low-fiber diet decreased levels of SCFAs and increased allergic airway inflammation [68]. Additionally, pregnant mice fed with the SCFA acetate had offspring with less allergic airway disease, but these effects were not present when the diet was only provided to lactating mice [74]. In contrast, a human study found that fiber intake during pregnancy was not associated with asthma/wheeze or sensitization in the offspring. However, pregnant women with higher fecal acetic acid were less likely to have offspring with wheeze or asthma [75]. Overall, this may indicate that the development of atopic diseases could take place already in utero.

Sandin et al. [76] examined fecal levels of SCFAs in young children and showed that at age 1 year, none of the SCFAs, including acetate, butyric, and propionic acid (among others), predicted sensitization at 4 years of age [76]. In contrast, a reduced level of fecal acetate was associated with wheeze and allergy in children and linked to an altered gut microbiome in a study by Arrieta et al. [35]. Butyrate level at age three months did not differ between children with wheeze and atopy compared to HC at age 1 year, whereas acetate was significantly reduced in children with wheeze and atopy [35]. Likewise, another study reported that 1-year-old children with high fecal butyrate and propionate levels were less likely to have asthma between 3 and 6 years and had significantly less allergic sensitization [77].

In conclusion, these findings highlight the role of SCFAs in the development of asthma and atopy, but further evidence is needed to investigate if SCFA-targeted treatment could be an effective preventive strategy.

### 3.5. PUFAs

Several studies have suggested PUFAs associated with asthma in children and adults [12,61,78,79,80,81]. In this review, one of the included studies showed that fecal PUFAs were inversely associated with asthma in children [31]. In addition, linoleic acid metabolism in plasma was significantly enriched and associated with lung function in asthmatic children [27]. The n-6 PUFAs are major precursors of proinflammatory eicosanoids through arachidonic acid and its metabolites. Arachidonic acid in membrane phospholipids is known to be implicated in airway inflammation and asthma pathogenesis via conversion to eicosanoids, leukotrienes, and prostaglandins [82,83,84], resulting in bronchoconstriction and inflammation. On the other side, n-3 PUFAs have anti-inflammatory effects and might prevent the development of asthma by antagonizing the effects of arachidonic acid [85]. It is also shown that some derivatives of dietary n-3 PUFAs are capable of displacing arachidonic acid from the cell membrane and dampen inflammation and hyperresponsiveness in asthmatic murine models [86,87].

Docosapentaenoate was the only significant PUFA metabolite identified in two of the included studies. This metabolite could distinguish children with asthma from children with food allergy [16], and a decreased level was associated with asthma [31]. Lower levels of n-6 PUFAs in asthmatics may suggest higher use of n-6 PUFAs in eicosanoid conversion in airway inflammation. On the other hand, a high n-6:n-3 ratio is thought to be pro-allergic [12]. However, results are inconsistent within and across biospecimens. One study suggested that arachidonic acid in plasma was 1.5-fold higher in asthmatic adults compared to controls [61]. In contrast, Magnusson et al. reported that arachidonic acid in plasma phospholipids in children at age eight years was inversely associated with prevalent asthma at age 16 [78].

PUFA levels across biospecimens may not relate to asthma in the same way. Lee-Sarwar et al. showed that intestinal PUFAs relate to asthma through bacterial dysbiosis, whereas plasma PUFAs drive different mechanisms in asthma [12]. PUFAs impact the fecal microbiome composition and have been associated with increased production of metabolites that influence asthma risk, such as the SCFA 12,13-dihydroxy-9-octadecenoic acid (12,13-diHOME) and conjugated linoleic acid (CLA). 12,13-diHOME is a relatively uncharacterized n-6 linoleic acid. Neonates at risk of childhood atopy and asthma exhibit higher fecal concentrations of 12,13-diHOME and perturbation of the gut microbiome with metabolic dysfunction [88]. However, the underlying mechanism is not known. Moreover, CLA consumption or microbial production has also been linked to increased intestinal SCFAs in murine studies [89], demonstrating a link between SCFAs and PUFAs.

Human gut microbes can metabolize PUFAs to produce CLAs [90], which, as supplementation itself, may improve control of existing asthma disease [12]. In an RCT with children aged 6–18 years with asthma and allergic sensitization, CLA supplementation was associated with decreased peripheral blood mononuclear cell production of IFN-gamma and IL-4. Still, it did not improve lung function or asthma symptoms [91]. In another RCT in overweight adults with mild asthma, CLA supplementation significantly improved airway hyperresponsiveness [92]. Finally, in an RCT including adults with pollen allergy, CLA supplementation significantly reduced sneezing during pollen season, and production of TNF-alfa, IFN-gamma, and IL-5 [93].

PUFAs have been investigated in children with allergy [94] and atopic dermatitis [95]. Korean children with allergic rhinitis and AD had lower n-3 PUFAs and higher n-6 PUFAs than children without atopic disease in blood samples. Further, arachidonic acid was significantly lower in subjects without atopic disease than those with [95]. In children with AD, a study suggested that dysregulation in n-6 PUFA metabolism may be associated with IgE production and atopy in general and not with AD [96].

The increased prevalence of atopic diseases parallels with a decrease in dietary intake of n-3 PUFAs and increased intake of n-6 PUFAs [95,97]. Exposure to n-6 and n-3 PUFAs during pregnancy might influence the risk of childhood wheeze and atopy [80]. In that context, fish-oil supplementation with n-3 LCPUFA in pregnancy was shown to reduce the risk of asthma in the offspring and affected the child’s metabolome [79] by downregulating the n-6 LCPUFA and tryptophan pathways and upregulating the tyrosine pathway. Another study found that n-6 PUFA intake and plasma levels of n-6 and n-3 PUFAs were inversely associated with asthma and/or recurrent wheeze at age three years [81]. Intake of oily fish or fish-oil supplementation during pregnancy is suggested to modify epigenetic programming in the offspring [98,99] and may be a strategy to prevent childhood atopic diseases [97].

In conclusion, further, particularly longitudinal, studies are needed to investigate the potential actions of n-3 and n-6 PUFAs on atopic inflammation. This could contribute to a better understanding of the biochemical mechanism of PUFAs on the development of asthma and other atopic diseases and the potential for targeted therapy and prevention.

### 3.6. Lipids

Lipids, particularly sphingolipids, were associated with asthma [16,19,25,27], food allergy [16], and AD [37] across the included studies, and lung function incentives were related to certain lipid sub-pathways. Kelly et al. reported glycerophospholipid-anchor biosynthesis significantly correlated with FEV1 [25]. The sphingolipid pathway was associated with airway hyperresponsiveness [27] and the glycerophospholipid pathway was associated with both airway hyperresponsiveness and the FEV1/FVC ratio at baseline and after bronchodilator [27].

Recent studies have linked childhood asthma to the orsomucoid-1-like protein 3 (ORMDL3) gene and the asthma susceptibility locus at 17q21. ORMDL3 inhibits the rate-limiting enzyme serine palmitoyl CoA transferase in the de novo sphingolipid synthesis [100], which results in decreased sphingolipid synthesis and increased airway hyperreactivity, independent of allergy or inflammation in mouse models [101]. In children aged 5–17 years, Ono et al. reported that the blood de novo sphingolipid synthesis was lower in children with asthma compared with controls [102]. Furthermore, decreased sphingolipids were found in children with high-risk variants in the 17q21 locus and children with non-allergic asthma compared to children with allergic asthma and HCs [102]. Moreover, children with non-allergic asthma had lower dihydroceramides, ceramides, and sphingomyelins compared to HCs. A lower level of sphingolipids has also been associated with a history of anaphylaxis [16]. In contrast, children with allergic asthma had higher dihydroceramides, ceramides, and sphingomyelins compared with children with non-allergic asthma. Lower sphingolipid synthesis is related to 17q21 variations, which are associated with asthma risk and higher ORMDL3 expression [102].

The de novo pathway of sphingolipid synthesis occurs in the endoplasmic reticulum and is the key mechanism for regulating cellular levels of ceramide and other sphingolipids [103]. Sphingolipids are the key structure in cellular membranes and act as signaling molecules involved in several functions: immune response, signal transduction, cell proliferation, cell growth, differentiation, and apoptosis [101]. Sphingosine-1-phosphate and ceramide have been reported as important signaling molecules in airway hyperreactivity, mast cell activation, and inflammation [101]. Ceramides act as a substrate for the production of more complex sphingolipids, e.g., sphingomyelin and glycosphingolipids [101]. In asthmatics adults, Sun et al. demonstrated a significantly decreased expression of the sphingosine-1-phosphate receptor in pulmonary vessels in asthmatic lungs compared to non-asthmatic individuals [104]. Sphingosine-1-phosphate appears to promote asthma, but plasma levels of the metabolite are also correlated with allergy to house dust mite in adults [105]. Specific ceramide species in serum were also elevated among asthmatic children with exercise-induced bronchoconstriction compared to those without [106].

In conclusion, sphingolipid metabolism seems to play a crucial role in childhood asthma pathogenesis and may be a target for future asthma therapeutics.

### 3.7. Strengths and Limitations

The metabolome is a snapshot of the biology and is influenced by genotype and environmental factors, e.g., age, sex, diet, height, weight, circadian rhythm, exercise, and medication. Many of the included studies in this review did not take these factors into account in their analyses. Some of these factors may mask the metabolic effects on atopic outcomes. Furthermore, the number of detected compounds depends on the profiling technique (NMR or MS) and the type of biospecimen investigated, making it challenging to compare results across the included studies. Liquid or gas chromatography combined with MS offers higher sensitivity, allowing measurement of a larger number of metabolites but with lower reproducibility compared to NMR. [13]. In this review, the majority of the selected studies used MS due to broader metabolite coverage.

Most of the included studies were based on urine samples, the second most was blood in either plasma or serum samples, and the third most used was stool samples. Urine is a stable biofluid that reflects both endogenous and exogenous metabolites and differs by age, sex, and collection season. Therefore, urine samples give an integrative view of both physiologic and environmental metabolites. Blood is composed of mostly endogenous metabolites and provides an instantaneous metabolite status serving as maintaining normal homeostasis through constant regulatory mechanisms [107]. Additionally, blood contains lipid-soluble metabolites that are not present in urine [38]. Urine and stool as biospecimen are non-invasive, easy to collect, and abundant for repeated collection. In contrast, blood sampling is invasive and less abundant for repeated pediatric collection.

Comparing metabolites in serum and plasma [108] has shown a generally higher serum concentration, which may be why serum demonstrates a higher sensitivity in biomarker detection [109]. The disadvantages of urine and stool samples are that they require transport and storage on ice. Though dysbiosis in gut microbiota has been implicated in several lung diseases, including asthma and allergy in line with the gut–lung axis theory, it is complicated to distinguish nutrition, endogenous, and gut microbiota metabolites in stool [110]. However, blood and urine samples lack specificity to airway physiology [107].

Several specific metabolites were examined in different biospecimens in the studies reviewed, but these examined different outcomes of atopic diseases. It is possible that the same specific metabolite can result in different findings from different biospecimens. However, in this review, we were unable to examine that.

The systematic search strategy applied for this review is a major strength. Furthermore, this review provides a comprehensive overview of metabolomics studies of asthma, allergy, or AD among children with a pathway-specific focus on the metabolism of tyrosine and tryptophan, lipids, PUFAs, SCFAs, and bile acids. A considerable limitation in the included studies is very few cohort studies or longitudinal studies. Most were case-control studies with small sample sizes, and only two [25,26] used a validation group to test if results could be replicated. The majority examined asthma/wheeze and fewer allergy or AD. Several studies did report the prevalence of AD, allergy, and allergic sensitization. Still, they did not take these atopic diseases into account in their analysis of metabolites and pathways related to asthma. Only one study [16] compared children with food allergy with and without asthma and found no difference in the levels of identified metabolites, suggesting a metabolomic signature concordant with that of food alone rather than asthma alone. This highlights that asthma-related metabolites and implicated pathways in asthmatic children may not solely reflect asthma status but may also influence metabolite profiles of other atopic traits in the same individuals.

Furthermore, asthma is a heterogeneous disease with various traits and different severities. Physician-diagnosed asthma was used in most of the included studies, but the definition of asthma diagnosis was not the same across studies. Some defined asthma according to international guidelines (GINA), including reversibility after bronchodilator. Others were based on history or use of asthma medication, physician diagnosed, or parental report of asthma or wheezing. In some studies, asthmatic children were defined as having “stable asthma” or “clinical asthma” or just “physician-diagnosed asthma” without further details of the confirmation of diagnosis. Put together, this raises the issue that the results may be influenced by these different definitions of asthma and calls for standardization in diagnosing asthma in future studies.

## 4. Materials and Methods

First, an initial limited search in the PubMed database was undertaken to identify keywords and MeSH terms related to metabolomics, types of biospecimen, children, and atopic conditions: asthma, wheeze, AD, allergic rhinitis, or food allergy. Second, a systematic search string was conducted by combining blocks of identified keywords and MeSH terms related to ‘atopy’ (asthma, allergy or AD), ‘metabolomics’, and ‘children’ presented in the Supplementary Materials (Table S2). No time restriction was applied to the PubMed search. Types of biospecimen limited the number of hits and were therefore not included in the search string.

The database search was finalized by the end of June 2020. The inclusion criteria were: (1) Age of children ≤18 years; (2) asthma, wheeze, allergic rhinitis, food allergy, or AD; (3) analytical platform: Mass spectrometry (MS) or nuclear magnetic resonance spectroscopy (NMR); (4) ≥ five metabolites reported; and (5) original studies published in English. We excluded studies that included animals or adults and studies examining metabolomics in EBC or VOC. Moreover, ongoing studies, not original studies, and studies that considered other topics than the examined were excluded.

The Human Metabolome Database, PubChem database, and KEGG PATHWAY databases were used to check if the reported metabolites belonged to the prespecified pathways of interest.

## 5. Conclusions

This review provides a detailed overview of the existing evidence from metabolomics studies of asthma, allergy, and AD in children with a pathway-specific focus on the metabolism of tyrosine and tryptophan, lipids, PUFAs, microbial derivatives, and bile acids. Several metabolites and pathways measured from different biospecimens were found to be associated with childhood atopic disease, particularly asthma, across the included studies, most of which in urine that is easy to collect in a pediatric setting. However, more studies are needed to investigate the potential of identifying a number of metabolites for predicting atopic disease development, differentiating endotypes of disease, and developing targeted therapeutics to specific atopic disease endotypes.

## Figures and Tables

**Table 1:** Summary of results for the 25 metabolomic studies in children.

Biospecimen	Atopic Focus Age Group	Method, Metabolomic Profiling	No. of Metabolites	Results	Significant Metabolites within Pathways of Interest	Pathway of Interest	Validation
Urine [15],	Asthma 6–11 years old	GC-MS, untargeted	766 peaks. 223 identified	PLS-DA models on the basis of 72 and 13 metabolites respectively distinguished uncontrolled asthma from controlled asthma and from healthy controls (HC) (R2 = 0.85; Q2 = 0.75); AND uncontrolled asthma from controlled asthma (R2 = 0.96; Q2 = 0.82) 22 and 9 pathways respectively were relevant to uncontrolled and controlled asthma	Tyrosine Tryptophan Hydroxyphenyllactic acid 3-hydroxyphenylacetic acid 5-hydroxyindoleacetic 4-hydroxybutyric acid 3-hydroxybutyric acid	Tyrosine metabolism Tryptophan metabolism Microbial derivatives (SCFAs)	Internal validation
Urine [20]	Asthma 6–17 years old	LC-MS, untargeted	8.570 variables	2-way hierarchical cluster analysis (HCA) determined 30 metabolites (at FDR q = 0.05) showing different levels between corticosteroid responders and corticosteroid nonresponders	3,4-dihydroxy-L-phenylalanine 3-Methoxy-4-hydroxyphenyl(ethylene)glycol)	Tyrosine metabolism	No
Urine [36]	Atopic dermatitis 6–10 months old	NMR, untargeted	Minimum 10 identified	The supervised canonical analysis identified 10 metabolites with different levels in atopic dermatitis and HC	2-hydroxybutyrate	Microbial derivatives (SCFAs)	Internal validation
Urine [18]	Asthma, allergy 1–4 years old	NMR, untargeted	76	PLS-DA models showed a clear separation between asthmatics and HC with a significant permutation test at age 1 and 2 years. Four metabolites were significantly associated with childhood asthma development in longitudinal analysis	N-acetyltyrosine	Tyrosine metabolism	Internal validation
Urine [21]	Asthma 7–17 years old	LC-MS, untargeted	6744 variables	OPLS-DA models on the basis of 40, 72 and 27 variables respectively discriminated asthmatics from HC (R2 = 0.85; Q2 = 0.75); asthmatics with controlled and poorly controlled symptoms using controller drugs from asthmatics with well-controlled asthma without using controller drugs (R2 = 0.81; Q2 = 0.72); AND asthmatics with well-controlled symptoms using controller drugs from asthmatics with poorly controlled symptoms using controller drugs (R2 = 0.88; Q2 = 0.76)	None	None	Internal validation
Urine [22]	Asthma 5–12 years old	GC-MS, targeted	34 organic acids	Statistically significant correlations were found between 5 metabolites and conventional pulmonary diagnostic tests (spirometry OR exhaled nitric oxide OR peak expiratory flow) in asthmatic children.	5-hydroxyindoleacetic acid 4-hydroxyphenylacetic acid	Tryptophan metabolism Tyrosine metabolism	No
Urine [24]	Asthma 4–16 years old	NMR, targeted	70	PLS-DA models on the basis of 23, 28 and 30 metabolites respectively distinguished stable asthma from HC (sensitivity, 94%; specificity, 95%; R2 = 0.72; Q2 = 0.67) AND stable from unstable asthma (R2 = 0.84; Q2 = 0.74) AND stable asthma from unstable asthma and from HC (R2 = 0.74; Q2 = 0.61)	Tyrosine Tryptophan 2-hydroxyisobutyrate	Tyrosine metabolism Tryptophan metabolism Microbial derivatives (SCFAs)	Internal validation
Urine [33]	Wheeze Infants followed up at age 1, 2, 3 years old	NMR, untargeted	Minimum 21 identified	None of the metabolites were significantly different between infants with respiratory syncytial virus (RSV) and human rhinovirus (HRV) infection after adjusting FDR for multiple testing. Alanine was the only metabolite associated with reduced risk of 1st-year-wheezing after adjusting FDR for multiple testing	None	None	Internal validation
Urine [19]	Wheeze/asthma 2–5 years old	LC-MS, untargeted	3411, minimum 28 identified	VIP-based PLS-DA identified a subset of metabolomic variables that clearly distinguished the transient wheezing group from the early-onset asthma group. The model showed an AUC = 0.99 and an AUC = 0.88 on sevenfold full cross-validation (*p* = 0.002)	Indole Glutaric acid 5-hydroxy-1-tryptophan Indole-3-acetamide Indolelactic acid p-Cresol Phosphatidyl glycerol 3-indoleacetic acid	Tryptophan metabolism Microbial derivatives Lipids	Internal validation
Urine [30]	Asthma 4 weeks-7 years old	UPLC-MS, untargeted	1555 Rt_*m*/*z* variables	Univariate analysis showed different levels of 63 and 87 features (q-value < 0.15) in the cohorts, respectively, between asthmatic and HC. 14 metabolites were common among the cohorts. Multivariate VIP-based PLS-DA and random forest analysis confirmed the discriminatory capacity of the metabolic profiles in both cohorts	Taurochenodeoxycholate-3-sulfate	Bile acids	Internal validation
Urine [32]	Wheeze Infants followed up to age 2 years old	LC-MS, untargeted	1588 variables	Univariate and multivariate analyses based on 30 identified metabolites discriminated children with postbronchiolitis wheezing from those who did not experience wheezing episodes. Pathway overrepresentation analysis pointed to a major involvement of the citric acid cycle and some amino acids; *p* ≤ 0.015)	None	None	Internal validation
Urine [35]	Wheeze, allergy 3–12 months old	UPLC-MS and GC-MS, untargeted	580 identified	Univariate tests showed that levels of 39 metabolites differed significantly at 3 months, and levels of 28 metabolites differed significantly at age 1 year between children with a 1-year phenotype of wheeze and allergy compared to HC	Glycolithocholate sulfate Glycocholenate sulfate Glycohyocholate Tauroursodeoxycholate	Bile acids	No
Urine [23] Serum [23]	Asthma 1–12 years old	NMR, untargeted	Minimum 36 identified	OPLS models for the classification of the control group and combination treatment group obtained a satisfactory validation in both urine and serum (R2 (cum) = 0.99; Q2 (cum) = 0.99). 21 metabolites in urine and 22 metabolites in serum significantly discriminated the two groups. 7 metabolic pathways were altered	Urine: 5-hydroxyindoleacetic acid Serum: 5-hydroxyindoleacetic acid Taurine 3-hydroxybutyrate 4-hydroxybutyrate	Microbial derivatives (SCFAs) Tryptophan metabolism Bile acids	Internal validation
Urine [38] Plasma [38]	Asthma, allergy 3–5 years old	NMR, untargeted	34 known in plasma and 44 known in the urine.	VIP-based PLS-DA based on 12 metabolites in plasma and 10 metabolites in urine significantly either discriminated children with asthma from controls OR discriminated children with and without mite, food, and IgE sensitization	Urine: N-acetyltyrosine Tyrosine Plasma: Acetic acid	Tyrosine metabolism Microbial derivatives (SCFAs)	Internal validation
Plasma [25]	Asthma 6–14 years	LC-MS, untargeted	8185 metabolite features. 574 known metabolites	8185 metabolite features were clustered into eight metabolite modules, where six were associated with lung function (*p* ≤ 0.05). The metabolite modules were enriched for lipid and amino acid metabolism	None (Significant subpathway)	Lipids	External validation (CAMP)
Plasma [26] (fasting)	Asthma 6–10 years old	UPLC-MS and GC-MS, untargeted	345 identified metabolites	None of the tested metabolites could significantly discriminate asthmatics from controls after adjustment for multiple comparisons. The PLS-DA model was not robust after seven-fold internal cross-validation. (R2 = 0.25; Q2 = 0.05). This was confirmed by the permutation testing (*p* = 0.134)	p-cresol sulfate Taurocholate Biliverdin	Bile acids Microbial derivatives	Internal validation External validation (VDAART)
Plasma [27]	Asthma 6–14 years old	LC-MS, untargeted	8185 metabolite features. 574 known metabolites	PLS-DA models based on all metabolites showed poor discriminatory ability after seven-fold internal cross-validation (R2 and Q2 < 0.1 for all three endpoints). FEV1/FVC ratios performed slightly better than airway hyperresponsiveness (AHR), and permutation testing confirmed that these models were robust. AHR was associated (*p* < 0.05) with 91 of 574 metabolites (15.9%), FEV1/FVC pre-bronchodilator with 102 (17.8%), and FEV1/FVC post-bronchodilator with 155 (27.0%).	None (Significant subpathway)	PUFAs Lipids	Internal
Plasma [28]	Asthma 6–17 years old	LC-MS, untargeted	8953	Unsupervised metabolomic analysis identified 164 metabolites that differed significantly between mild-to-moderate asthma vs. severe refractory asthma (FDR of 0.01). The metabolites were significantly associated with 2 metabolic pathways: the glycine, serine, and threonine metabolism pathway and the N-acylethanolamine and N-acyltransferase	None	None	No
Plasma [14]	Asthma 1–18 years old	LC-MS targeted lipids	64 metabolites. 32 identified	The metabolite concentrations were not significantly different between subjects, with or without β-2-agonist use, after adjusting for multiple testing. None of the pathways were significantly associated with asthma control.	None	None	No
Serum [29]	Asthma 9–19 years old	LC-MS, targeted	308 selected metabolites 30 lipid mediators	Univariate tests showed that 14 metabolites significantly discriminated children with and without asthma. There were no differences in lipid mediators by asthma status after Bonferroni correction.	Shikimate-3-phosphate	Tryptophan metabolism Tyrosine metabolism	Internal validation
Serum [37] (fasting)	Atopic dermatitis 3 months-3 years old	LC-MS, untargeted and targeted eicosanoids	Untargeted: NR 30 targeted eicosanoids	OSC-PLS-DA models clearly showed the separation of children with AD with high IgE levels and HC and children with AD with normal IgE children and HC. Based on targeted eicosanoids, HC were separated from AD children with normal or high IgE levels.	Untargeted: Indolelactic acid Tryptophan Sphingomyelins: SM 34:2, SM 36:2 SM(d18:1/16:1)(OH) Glycocholic acid Glycochenodeoxycholic acid (GCDCA) Taurocholic acid (TCA) Taurochenodeoxycholic acid (TCDCA) Cholic acid (CA) Chenodeoxycholic acid (CDCA) Targeted: 8, 9, 11, 16, 19, 20- HETE 9-, 13-HODE	Tryptophan metabolism Tyrosine metabolism Lipids Bile acids PUFAs	Internal validation
Serum [16]	Asthma, food allergy 1–12 years old (cases). Controls up to 18 years old	UPLC-MS, untargeted	1165 compounds. 868 identified	There were no differences in the levels of the 81 identified metabolites when comparing children with food allergy with or without asthma. There were no differences in levels of the 53 identified metabolites between children with food allergy with and without AD. In conclusion, children with food allergy exhibited a disease-specific metabolomic signature	3-(4-hydroxyphenyl)lactate 3-methoxytyramine sulfate, 4-methoxyphenol sulfate Phenol glucuronide Phenol sulfate 5-hydroxyindoleacetate Taurochenodeoxycholate Taurocholate Glycocholate Tauroursodeoxycholate Glycohyocholate, Glycoursodeoxycholate Taurohyocholate Dihomo-linolenate (20:3n3 or n6) Docosapentaenoate (n6 DPA; 22:5n6) Quinolinate Arachidonate	Tyrosine metabolism Tryptophan metabolism Bile acid metabolism Lipid metabolism PUFA metabolism	No
Stool [17]	Asthma, allergy 4–7 years old	NMR, untargeted	40 known metabolites	PLS-DA models were not robust after 10-fold cross-validation (R2 >> Q2 for asthma vs. HC, rhinitis vs. HC, AND asthma vs. rhinitis vs. HC. This was confirmed by permutation testing (permuted *p* = 0.27–0.78). Univariate tests showed that 1 and 5 metabolites, respectively, discriminated children with rhinitis from HC AND asthmatics from HC (FDR-adjusted *p*-value < 0.05)	Butyrate 4-hydroxybutyrate	Microbial derivatives(SCFAs)	Internal validation
Stool [31]	Asthma 3 years old	MS, untargeted	737 annotated metabolites	Adjusted logistic regression analyses identified 45 metabolites significantly associated with asthma at age 3 (*p* < 0.05). A total of 5 of the 45 metabolites remained significant after correcting for multiple testing. Modules of highly correlated asthma-associated lipid metabolites included PUFAs, endocannabinoids, and diacylglycerols	p-cresol sulfate Docosapentaenoate	Microbial derivatives PUFAs	No
Amniotic fluid [34]	Wheeze Unborn, follow up at age 1 year	LC-MS and LC-MSE, untargeted	1706	16 metabolites with a plausible biological significance discriminated infants with wheeze during the first year of life from HC. Pathway analysis demonstrated that 5 of these variables are part of 2 pathways: steroid hormone biosynthesis (*p* = 0.003) and 2-phenylalanine metabolism (*p* = 0.009) emerging as probably perturbed pathways.	5-hydroxyindolepyruvate 3-Hydroxyphenylacetic acid p-cresol glucuronide	Tryptophan metabolism Tyrosine metabolism Microbial derivatives	Internal validation

Abbreviations: PLS-DA: Partial Least Squares Discriminant Analysis; OPLS-DA: Orthogonal Projections to Latent Structures Discriminant Analysis; OSC: orthogonal signal correction; VIP: Variable Influence on Projection; AUC: Area Under the Curve; MS: mass spectrometry; LC: liquid chromatography; GC: gas chromatography; NMR: nuclear magnetic resonance; UPLC: Ultra performance liquid chromatography; HC: healthy controls; AD: atopic dermatitis; FDR: false discovery rate; FEV1: Forced Expired Volume in the first second; FVC: Forced Vital Capacity; RSV: respiratory syncytial virus; HRV: human rhinovirus. Q2: goodness of prediction; R2; model’s fit of the data; VDAART: The Vitamin D Antenatal Asthma Reduction Trial; CAMP: Childhood Asthma Management Program.

**Table 2:** Metabolites within pathways of interest identified as significant in more than one study.

Metabolite/Metabolite Derivatives	Pathway of Interest	Biospecimen	Method	Age Group	Atopic Focus	Outcome
Tyrosine	Tyrosine metabolism	Urine [15,24,38]	GC-MS [15] NMR [24,38]	6–11 years old [15] 4–16 years old [24] 3–5 years old [38]	Asthma [15,24] Food allergy [38]	↑ in uncontrolled asthma vs. healthy controls (HC) [15] ↑ in uncontrolled asthma vs. controlled asthma [15] Distinguish unstable asthma from stable asthma [24] Distinguish stable asthma from HC [24] ↑ in food sensitization (egg or milk) vs. no food sensitization [38]
3-hydroxyphenylacetic acid [15,34] Hydroxyphenyllactic acid [15] 4-hydroxyphenylacetic [22]	Tyrosine metabolism	Urine [15,22] Amniotic fluid [34]	GC-MS [15,22] LC-MS and LC-MSE [34]	6–11 years old [15] 5–12 years old [22] Unborn, follow up at age 1 year [34]	Wheeze [34]/Asthma [15,22]	↓ 3-hydroxyphenylacetic acid in asthma vs. in HC [15] ↑ in infants without wheezing in their first year compared to wheezing infants [34] ↑ Hydroxyphenyllactic acid in uncontrolled asthma vs. HC [15] Negative correlation with forced expired volume in the first second (FEV1) and forced vital capacity (FVC) [22]
N-acetyltyrosine	Tyrosine metabolism	Urine [18,38]	NMR [18,38]	1–4 years old [18] 3–5 years old [38]	Asthma [18] Food allergy [38]	↓ in children at age 1 who develop asthma before age 4 vs. HC [18] ↑ in food sensitization (egg or milk) vs. no food sensitization [38]
Tryptophan	Tryptophan metabolism	Urine [15,24] Serum [37]	GC-MS [15] NMR [24] LC-MS [37]	6–11 years old [15] 4–16 years old [24] 3 months-3 years old [37]	Asthma [15,24] Atopic dermatitis [37]	↑ in uncontrolled asthma vs. HC [15] Distinguish unstable from stable asthma and stable asthma from HC [24] ↑ in AD with high IgE vs. HC and vs. AD with normal IgE level [37]
Indolelactic acid	Tryptophan metabolism	Urine [19] Serum [37]	LC-MS [19,37]	2–5 years old [19] 3 months-3 years old [37]	Wheeze/asthma [19] Atopic dermatitis [37]	↑ Transient wheezing vs. early-onset asthma [19] ↑ in AD with high IgE vs. HC and vs. AD with normal IgE level [37]
5-hydroxyindoleacetic acid/ 5-hydroxyindoleacetate (5-HIAA)	Tryptophan metabolism	Urine [15,22,23] Serum [16,23]	GC-MS [15,22] NMR [23] UPLC-MS [16]	5–12 years old [22] 6–11 years old [15] 1–12 years old [16,23]	Asthma [22,23] Food allergy [16]	↑ with a combination treatment of budesonide and salbutamol during acute asthma exacerbation [23] Distinguish HC from uncontrolled asthma and controlled asthma [15] Positive correlation with FEV1/FVC and negative correlation with exhaled nitric oxide (eNO) [22] Distinguish asthma vs. food allergy [16]
butyrate [17] 4-hydroxybutyrate [17] 4-hydroxybutyric acid [15] 3-hydroxybutyrate [23] 3-hydroxybutyric acid [15] 3-hydroxyisobutyric [23] 2-hydroxyisobutyrate [24] 2-hydroxybutyrate [36]	SCFAs (Microbial derivatives)	Stool [17] Urine [15,24,36] Serum [23]	NMR [23,24,36] GC-MS [15]	4–7 years old [17] 4–16 years old [24] 6–11 years old [15] 6–10 months old [36] 1–12 years old [23]	Asthma [15,17,23,24] Atopic dermatitis [36]	↑ 4-hydroxybutyrate in asthma vs. HC and ↓ butyrate in asthma vs. HC [17] Distinguish stable asthma from HC [24] ↑ AD vs. HC [36] ↑ 3-hydroxybutyric acid in uncontrolled vs. controlled asthma, but ↓ in asthmatics vs. HC [15] ↓ 4-hydroxybutyric acid. controlled and uncontrolled asthma vs. HC [15] ↑ with a combination treatment of budesonide and salbutamol during acute asthma exacerbation [23]
p-Cresol sulfate [26,31] p-Cresol [19] p-Cresol glucuronide [34]	Microbial derivatives (through tyrosine metabolism)	Stool [31] Urine [19] Plasma [26] Amniotic fluid [34]	MS [31] LC-MS [19] UPLC and GC-MS LC-MS and LC-MSE [34]	3 years old [31] 2–5 years old [19] 6–10 years [26] Unborn, follow up at age 1 year [34]	Wheeze/asthma [19,26,31,34]	Inversely associated with asthma [31], ↑ in the “transient wheezing” vs. ”early-onset” asthma [19] associated with wheezing during the first year of life vs. controls without wheezing episodes [34] current asthma was associated with a reduced level of p-Cresol sulfate vs. HC. This association was replicated in the validation group [26]
Taurocholate/Taurocholic acid (TCA) [16,26,37]	Bile acids	Plasma [26] Serum [16,37]	UPLC-MS and GC-MS [26] UPLC-MS [16] LC-MS [37]	6–10 years old [26] 1–12 years old [16] 3 months-3 years old [37]	Asthma [16,26] Atopic dermatitis [37]	↑ associated with current asthma [26] Distinguish asthma from non-atopic controls [16] Distinguish food allergy from asthma after adjustment for AD [16] ↓ AD independently of high or normal IgE level vs. HC [37]
Taurochenodeoxycholate/ Taurochenodeoxycholic acid (TCDCA) [16,37] Taurochenodeoxycholate-3-sulfate [30]	Bile acids	Serum [16,37] Urine [30]	UPLC-MS [16,30] LC-MS [37]	1–12 years old [16] 3 months-3 years old [37] 4 weeks-7 years old [30]	Wheeze [30]/Asthma [16,30] Atopic dermatitis [37]	Distinguish asthma from non-atopic controls [16] ↓ AD independently of high or normal IgE level vs. HC [37] ↑ in children who developed persistent wheeze/asthma [30]
Glycohyocholate/Glycohyocholic acid (GHCA)	Bile acids	Serum [16] Urine [35]	UPLC-MS [16] UPLC-MS and GC-MS [35]	1–12 years old [16] 3–12 months old [35]	Asthma [16] Wheeze and atopy [35] Food allergy [16]	Distinguish food allergy from asthma after adjustment for AD [16] ↑ in those children who developed atopy and wheeze at age one year compared to controls [35]
Glycocholate/glycocholic acid (GCA)	Bile acids	Serum [16,37]	UPLC-MS [16] LC-MS [37]	1–12 years old [16] 3 months-3 years old [37]	Asthma [16] Atopic dermatitis [37]	Distinguish asthma from non-atopic controls [16] ↓ AD independently of high or normal IgE level vs. HC [37]
Docosapentaenoate n-6	PUFAs	Stool [31] Serum [16]	MS [31] UPLC-MS [16]	3 years old [31] 1–12 years old [16]	Asthma [16,31] Food allergy [16]	Inversely associated with asthma [31] Distinguish food allergy from asthma after adjustment for AD [16]

Abbreviations: MS: mass spectrometry; LC: liquid chromatography; GC: gas chromatography; NMR: nuclear magnetic resonance; UPLC: Ultra performance liquid chromatography; HC: healthy controls; AD: atopic dermatitis; IgE: immunoglobulin E; FEV1: Forced Expired Volume in the first second; FVC: Forced Vital Capacity.

**Table 3:** Pathways of interest associated with asthma, allergy, or atopic dermatitis in more than one study.

Pathway	Biospecimen	Method	Atopic Focus	Outcome
Tyrosine metabolism	Urine [15,18,20,22,24,38] Serum [16,29] Amniotic fluid [34]	GC-MS [15,22] NMR [18,24,38] LC-MS [20,29] LC-MS and LC-MSE [34] UPLC-MS [16]	Wheeze [34]/Asthma [15,18,20,22,24,29] Food allergy [16,38]	Significant metabolite(s) within pathway associated with wheeze, asthma [15,18,20,22,24,29,34] or food allergy [16,38]. A statistically significant asthma pathway [15]. Pathway affected by corticosteroid resistance [20].
Tryptophan metabolism	Urine [15,19,23,24] Serum [16,23,29] Amniotic fluid [34]	GC-MS [15] NMR [23,24] LC-MS [19,29,37] UPLC-MS [16] LC-MS and LC-MSE [34]	Wheeze/Asthma [15,19,23,24,29,34] Atopic dermatitis [37] Food allergy [16]	Significant metabolite(s) within pathway associated with wheeze [34], asthma [15,19,22,24,29], food allergy [16], atopic dermatitis [37] or asthma treatment [23]
Microbial derivatives Including (SCFAs)	Plasma [26] Stool [17,31] Amniotic fluid [34] Urine [15,19] Serum [23]	UPLC-MS and GC-MS [26] MS [31] LC-MS and LC-MSE [34] LS-MS [19] NMR [17,23,24,36] LC-MS [19] GC-MS [15]	Wheeze/Asthma [15,17,19,23,24,26,31,34] Atopic dermatitis [36]	Significant metabolite(s) within pathway associated with wheeze [19,34], asthma [15,17,24,26,31], AD [36] or asthma treatment [23]
Bile acids	Urine [30,35] Plasma [26] Serum [16,23,37]	UPLC-MS and GC-MS [26,35] UPLC-MS [16,30] LC-MS [37] NMR [23]	Wheeze/asthma [16,23,26,30,35] Atopic dermatitis [37] Food allergy [16]	Significant metabolite(s) within pathway associated with wheeze [35], asthma [16,26,30], food allergy [16], AD [37] or asthma treatment [23]. Pathway analysis revealed that one of the most dysregulated pathways associated with the presence of FA in comparison with asthma included secondary bile acid metabolism [16].
PUFAs	Plasma [27] Stool [31] Serum [16,37]	LC-MS [27,37] MS [31] UPLC-MS [16]	Asthma [16,27,31] Food allergy [16] Atopic dermatitis [37]	Significant metabolite(s) within pathway associated with asthma [16,31], food allergy [16] or AD [37]. Linoleic acid metabolism was significantly enriched and associated with three phenotypic aspects of asthma defined by the degree of lung function: airway hyperresponsiveness to methacholine and FEV1/FVC ratio before and after the use of a bronchodilator [27]. Hydroxyl octadecadienoic acids (HODEs) and most hydroxy eicosatetraenoic acids (HETEs) increased in AD (with high and normal IgE level) vs. controls [37]. PUFA module (including 9 PUFAs) was inversely associated with asthma [16].
Lipids, sphingolipids, and ceramides	Plasma [25,27] Urine [19] Serum [16,37]	LC-MS [19,25,27,37] UPLS-MS [16]	Asthma [16,25,27] Atopic dermatitis [37] Food allergy [16]	Significant metabolite(s) within pathway associated with asthma [16,19], food allergy [16] or AD [37]. Baseline FEV1 was significantly correlated with glycerophospholipid-anchor biosynthesis [25]. Enrichment in the glycerophospholipid pathway was associated with airway hyperresponsiveness and FEV1/FVC ratio before and after using a bronchodilator [27]. The sphingolipid pathway was specific airway hyperresponsiveness to methacholine [27]. Pathway analysis showed that the pathways strongest associated with the presence of FA in comparison with control subjects included dihydrosphingomyelins, lactosylceramides, sphingomyelins, and hexosylceramides, among others [16].

Abbreviations: MS: mass spectrometry; LC: liquid chromatography; GC: gas chromatography; NMR: nuclear magnetic resonance; UPLC: Ultra performance liquid chromatography; AD: atopic dermatitis; FA: food allergy; FEV1: Forced Expired Volume in the first second; FVC: Forced Vital Capacity.

## Supplementary Materials

The following are available online at https://www.mdpi.com/2218-1989/10/12/511/s1, Table S1: Characteristics of the 25 metabolomics studies conducted in children, Table S2: PubMed database search.
