# Supplementary material for: The Metabolomics of Childhood Atopic Diseases: A Comprehensive Pathway-Specific Review
Source: Metabolites. 2020 Dec 16;10(12):511. doi: 10.3390/metabo10120511 (PMC7767195; doi:10.3390/metabo10120511)
Supplement: Supplementary file 1 [file metabolites-10-00511-s001.pdf]

Supplementary materials

Table S1. Characteristics of the 25 metabolomics studies conducted in children.

Authors<br>Year<br>Country	Atopic focus<br>Age Group<br>Males, Total (%)	Biospecimen<br>Method<br>Metabolomic<br>Profiling	Study Design<br>Population<br>Ethnicity	No. of Cases	No. of Controls	Diagnostic criteria	Atopy	Use of Medication	Metabolomic Aim
Tao et. al.[15]<br>2019<br>China	Asthma<br>6–11 years old<br>75 (69)	Urine<br>GC-MS<br>Untargeted	Case-control<br>China<br>NR	37 with uncontrolled asthma<br>43 with controlled asthma	29 sex-matched healthy controls	PD, GINA guidelines	Allergy: Mite allergy was reported in 21 with uncontrolled asthma and 26 with controlled asthma. 8 cases were not tested. No mite allergy in controls.<br>AD: NR	31 cases with uncontrolled asthma and 37 cases with controlled asthma used inhaled corticosteroid (ICS) in the previous 3 days.	Metabolomic profile of asthma vs. healthy, and of different asthma endotypes
Park et al.[20]<br>2017<br>Korea and the USA	Asthma<br>6–17 years old<br>21(70)	Urine<br>LC-MS<br>Untargeted	Case-only<br>USA (Atlanta)<br>White/non-white: 6/24	30 with severe asthma:<br>15 corticosteroid (CS)-respondent<br>15 CS-nonrespondent	0	PD	Allergy and AD: NR	Children were treated with a high-dose inhaled corticosteroid and a second controller medication	Metabolomic profile of CS-respondent vs. CS-non-respondent asthma
Assfalg et al.[36]<br>2012<br>Italy	Atopic dermatitis<br>6–10 months old<br>17(53)	Urine<br>NMR<br>Untargeted	Case-control<br>Italy<br>NR	20 with AD	12 age-matched healthy controls	PD according to the United Kingdom working party's criteria	Allergic sensitization: 10 cases (50 %) with positive SPT to cow milk and/or egg allergy. Controls: NR	NR	Metabolomic profile of AD vs. healthy
Chiu et al.[18]<br>2018<br>Taiwan	Asthma, allergy<br>1–4 years old<br>22 (37)	Urine<br>NMR<br>Untargeted	Nested case-control<br>Taiwan<br>NR	30 diagnosed with asthma at age 4 years.	30 healthy children without asthma or other atopic conditions	PD<br>Allergen-specific IgE: sensitization was defined as values ≥0.35 kU/L	Children with rhinitis or eczema were excluded.<br>Mite, food, and IgE sensitization and total IgE levels are reported at age 3 years old.<br>AD: NR	NR	Identification of metabolic mechanisms underlying asthma development
Mattarucchi et al.[21]<br>2012<br>Italy	Asthma<br>7–17 years old<br>33 (62)	Urine<br>LC-MS<br>Untargeted	Case-control<br>Italy<br>All Caucasian	41 atopic asthmatics:<br>14 with well-controlled symptoms without daily controller drugs and 16 under daily controller	12 age-matched healthy controls with no history of atopy or respiratory diseases.	PD, GINA guidelines	Allergy and AD: NR	Well-controlled symptoms with use of β-2-agonist as needed (n=14)<br>or use of at least one daily controller drug (n=16).	Metabolomic profile of asthma vs. healthy, and of different asthma endotypes

				drugs.<br>11 with poorly controlled symptoms despite daily controller drugs and with at least two exacerbations requiring oral CS in the last year				Poorly controlled asthma with at least two daily controller drugs: (n=11)<br>AD: NR	
Papamichael et al.[22]<br>2019<br>Australia	Asthma<br>5–12 years old<br>NR	Urine<br>GC-MS,<br>Targeted	Case-only<br>Greece<br>NR	65 with mild asthma	0	PD	Allergy and AD: NR	The majority were taking asthma medication during the past month	Investigate possible relationships between urinary organic acids and pulmonary diagnostic tests
Saude et al.[24]<br>2011<br>Canada	Asthma<br>4–16 years old<br>86 (64)	Urine<br>NMR<br>Targeted	Case-control<br>Canada<br>NR	73 with stable asthma, 20 with unstable asthma in the emergency department	42 age- and sex-matched healthy controls	PD<br>Cases were considered atopic on the basis of at least 1 positive SPT to a panel of common aeroallergens	Allergic sensitization: 73% of cases (n=68) were atopic: n=55 with stable asthma and n=13 with unstable asthma. NR for controls<br>AD: NR	Use of ICS in n=51 with stable asthma and n=9 with unstable asthma	Metabolomic profile of asthma vs. healthy, and of different asthma endotypes
Turi et al.[33]<br>2019<br>USA	Wheeze<br>Infants followed up at age 1, 2, and 3 years old<br>52 (37)	Urine<br>NMR<br>Untargeted	Nested case-control<br>USA<br>White/Black/ Hispanic/Other (%): 69/13/11/7	80: 70 with respiratory syncytial virus infection (RSV) and 10 with human rhinovirus (HRV)	60 healthy controls	Recurrent wheeze defined as ≥ 3 wheezing events in the last year, or wheeze with the use of asthma medications in the past year based on parental report	Allergy and AD: NR	NR	Metabolomic profile of RSV vs. HRV infection, and of who do and do not wheeze in early childhood after respiratory infection in infancy
Carraro et al.[19]<br>2018<br>Italy	Wheeze/asthma<br>2–5 years old<br>NR	Urine<br>LC-MS<br>Untargeted	Case-control<br>Italy<br>NR	32 with recurrent wheezing. During the 3 years follow up: 16 were classified with transient wheezing, and 16 were classified with early-onset asthma	13: with no history of allergic or respiratory diseases. No wheezing symptoms during follow up.	PD, GINA. Early-onset asthma was defined as an experience of recurrent asthma-like symptoms during the past year and use of daily ICS for at least 6 months.<br>SPT	Early-onset asthma group: 11 with allergic sensitization, 3 with FA, and 3 with AR.<br>Transient wheezing group: 4 with allergic sensitization, 1 with FA, and 0 with AR.<br>AD in 4 with early-onset asthma and 2	Early-onset asthma: 5 used ICS and 3 used Montelukast.<br>Transient wheezing group: 3 used ICS and 2 used Montelukast.	Metabolomic profile of transient wheezing vs.early-onset asthma

							with transient wheezing group<br>Allergy and AD in controls: NR		
Chawes et al.[30]<br>2019<br>Denmark/Italy	Asthma<br>4 weeks–7 years old<br>NR	Urine<br>UPLC-MS<br>Untargeted	Two cohorts (COPSAC)<br>Denmark<br>NR	20 and 49 in the two cohorts respectively developed wheeze/asthma during the first 6 years of life	151 and 112 healthy children in the two cohorts, respectively, did not develop wheeze/asthma during the first 6 years of life	PD. Persistent wheeze (0–3 y)/asthma (>3 y) was diagnosed based on a validated quantitative symptom-based algorithm.	Allergy and AD: NR	No use of medication before urinary samples were collected	Development of persistent wheeze/asthma in the first 6 years of life in children born to mothers with asthma
Barlotta et al.[32]<br>2019<br>Italy	Wheeze<br><1 year old. Follow up until 2 years after an episode of bronchiolitis<br>49 (64)	Urine<br>LC-MS<br>Untargeted	Nested case-control<br>Italy<br>NR	Of 52 infants with bronchiolitis, 17 developed recurrent wheezing, 11 experienced 1 or 2 episodes of wheezing, and 24 did experience wheezing episodes during the 2 years of follow up	24 healthy infants with no history of bronchiolitis < 1 year	PD. Recurrent wheezing was defined as ≥3 episodes of	Allergy: NR<br>AD: in 11 cases (21%: 4 with no wheezing, 6 with recurrent wheezing, 1 with 1 of 2 wheezing episodes. NR for controls	NR	Metabolomic profile of infants with acute bronchiolitis who will subsequently develop recurrent wheezing from those who will not.
Arrieta et al.[35]<br>2015<br>Canada	Wheeze, allergy, 3–12 months old<br>21 (60)	Urine<br>UPLC-MS and GC-MS, Untargeted	Nested case-control<br>Canada<br>NR	19 with wheeze and allergy	16 healthy controls	Wheeze and AD are either diagnosed by clinicians or non-clinicians by questionnaire during the first year of life. SPT: classified as atopic if the development of a wheal ≥2 mm for any of 10 specific allergens occurred.	Allergic sensitization: All cases were atopic.<br>AD: 3 cases (16%) and 1 control (6%) at age 3 months and 11 cases (58%) and 8 controls (50%) at age 1 year old.	8 cases (48%) and 1 control (6%) used antibiotics during the first year of life.	Metabolic profile of infants with wheeze and allergy vs. healthy infants
Quan-Jun et al.[23]<br>2017<br>China	Asthma<br>1–12 years old<br>56 (48)	Urine and serum<br>NMR<br>Untargeted	Case-control<br>China<br>NR	69 inpatient children with asthma during acute exacerbation without the usage of any topical or systemic CS or β-2-agonist treatment in the previous 3 months	48 age-and sex-matched asthmatic controls with the usage of asthma medication.	PD	Allergy and AD: NR	During the study, a total of 37, 31, and 47 cases used procaterol, montelukast, and antibiotics, respectively. 31 controls were treated with antibiotics.	Metabolomic profile of combined treatment with ICS and β-2-agonist of children with asthma during acute exacerbation

Chiu et al.[38]<br>2020<br>Taiwan	Asthma, allergy<br>3–5 years old<br>35 (65)	Urine and plasma<br>NMR<br>Untargeted	Case-control<br>NR<br>NR	28 with asthma	26 healthy children without atopic conditions	PD: based on the guidelines of the Global Initiative for Asthma.<br>Serum total IgE.<br>Allergic sensitization is defined as allergen-specific IgE levels ≥ 0.35 kU/L to any of the 4 allergens.	Allergic sensitization:<br>Mite allergy: 19 cases (68%) and 5 controls (19%).<br>Food allergy: 15 cases (54%) and 7 controls (27%)<br>IgE>100 kU/L: 14 cases (50%) and 2 controls (8%)<br>AD: NR	NR	Metabolomic profile of asthma vs. healthy and children with mite, food, and IgE sensitization vs. without sensitization
Kelly et al.[25]<br>2018<br>USA	Asthma<br>6 to 14 years<br>190 (59)	Plasma<br>LC-MS<br>Untargeted	Case-only<br>Costa Rica<br>All Hispanic/Latino	325 with mild-to-moderate asthma	0	PD: ≥ 2 episodes of respiratory symptoms or asthma attacks in the prior year.	Allergy and AD: NR	297 (91,4 %) used controller treatment: either oral or inhaled steroids, prednisone, long-acting inhaled β2-agonists, or leukotriene inhibitors/modifiers	Identify metabolites associated with asthmatic lung function
Kelly et al.[26]<br>2018<br>USA	Asthma<br>6-10 years old<br>113 (48)	Plasma<br>UPLC-MS and /or GC-MS,<br>Untargeted	Nested case-control<br>USA<br>White/Black/Ot-her (%)<br>57/24/19	46 with current asthma	191 without asthma	Maternal report of ever diagnosed with asthma by a healthcare professional and either taking asthma medications or experienced wheezing symptoms in the past year.<br>Allergen sensitization was defined as any specific IgE level ≥ 0.35 IU/ml to common allergens	Allergen sensitization:<br>215 of 237 were tested.<br>Sensitization to any common allergen was found in 28 cases (61%)<br>and in 86 controls (45%)<br>AD: NR	The majority (80%) of children with current asthma were taking ICS.	Metabolomic profiles of asthma vs. healthy
Kelly et al.[27]<br>2017<br>USA	Asthma<br>6-14 years old<br>226 (60)	Plasma<br>LC-MS<br>Untargeted	Case-only<br>Costa Rica<br>All Hispanic	380: The majority of children were defined as mild to moderate asthmatics	0	PD: ≥ 2 respiratory symptoms or asthma attacks in the prior year.	Allergy and AD: NR	346 (91%) were on some form (inhaled or oral) of regular controller treatment	Metabolomic profile of asthmatic children by their degree of lung function
Fitzpatrick et al.[28]<br>2014<br>USA	Asthma<br>6-17 years old<br>35 (61)	Plasma<br>LC-MS<br>Untargeted	Case-only<br>USA (Atlanta)<br>Nonwhite: 48 (84%)	22 with mild to moderate asthma	0	PD. Total serum IgE.	AR: 91% of mild-to-moderate asthma (n=20) and 100% of	Severe refractory asthma: use of high-dose ICS and long-acting β-2-agonist.	Metabolomic profile of mild to moderate

				35 with severe refractory asthma			severe refractory asthma (n=35).<br>AD: 50% of mild-to moderate asthma (n=11) and 60% of severe refractory asthma (n=21)	Mild-to-moderate asthma: use of ICS or ICS/long-acting β-2-agonist combination therapy.	asthma vs. severe refractory asthma
McGeachie et al.[14]<br>2015<br>USA	Wheeze, asthma<br>1-18 years old<br>13 (65)	Plasma<br>LC-MS<br>Targeted	Case-only<br>USA<br>100 % self-reported European ancestry	8 with use of β-2-agonist and 12 with no use of β-2-agonist in the week preceding<br>blood is drawn	0	PD. Self-reported use of β-2-agonist inhalers in the week preceding blood drawn.	AR: 7 with use of β-2-agonist (88%) and 6 without (50%)<br>FA: 2 with use of β-2-agonist (25%) and 4 without (33%.<br>Eczema: 5 with use of β-2-agonist (63%) and 5 without (42%)	Use of ICS in the past week: 50% in the children who used β-2-agonist (n=4) and 42 % in the group who did not (n=5)	Metabolic profile of asthmatics with use of β-2-agonist vs. without
Checkley et al.[29]<br>2016<br>USA	Asthma<br>9-19 years old<br>57 (57)	Serum<br>LC-MS<br>Targeted	Nested case-control<br>Peru<br>NR	50 with current asthma	49 with no prior history of asthma and with normal lung function.	PD or self- or parental-report of wheezing or use of asthma medications in the past year.<br>Atopy: the presence of IgE antibodies to any of three allergen panels.	Allergic sensitization: 42 cases and 27 controls<br>AD: NR	4 out of 48 used ICS, and 12 out of 48 used β-2-agonist in the prior year. Data is missing in 2 cases.	Metabolic profile of asthma vs. healthy.
Huang et al.[37]<br>2014<br>China	Atopic dermatitis<br>3 months-3 years old<br>40 (62)	Serum<br>LC-MS,<br>Untargeted and targeted	Case-control<br>China<br>NR	19 AD patients with high IgE levels<br>23 AD patients with normal IgE levels	23	AD was diagnosed according to Hanifin and Rajka diagnostic criteria.	Controls: all with normal IgE levels	NR	Metabolic profile of AD vs. healthy and of children with AD high vs. normal IgE levels
Crestani et al.[16]<br>2019<br>USA	Asthma, allergy<br>1-12 years old (cases). Controls up to 18 years old<br>70 (56)	Serum<br>UPLC-MS<br>Untargeted	Case-control<br>USA<br>White/Hispanic/ African American/Asian/Other or not available: 51/33/27/5/9	35 with asthma: intermittent, mild persistent or moderate persistent<br>35 with FA<br>35 with both asthma and FA	20 controls without AD or FA	Asthma diagnosis was based on a validated questionnaire and American Thoracic Society criteria.<br>The diagnosis of FA was made by the	AR: in 27 with asthma (77%), in 23 with FA (67%), and in 30 with both asthma and FA (86%).<br>AD: in 8 with asthma (23%), in 21 with FA (60%), and in 20 with	NR. However, exclusion criteria were use of steroids, immunosuppressive medications, and use of antibiotics within the previous 6 weeks	Metabolomic profile of asthma vs. healthy and of FA with/without asthma vs. healthy.

						treating physician and was based on a combination of positive IgE test results (SPT, specific IgE, or both)	both asthma and FA (57%).		
Chiu et al.[17] 2019 Taiwan	Asthma, allergy 4-7 years old 50 (59)	Stool NMR Untargeted	Case-control Taiwan 100 % same ethnic and geographical region	34 with asthma 27 with rhinitis	24 healthy controls without a history of asthma or other atopic conditions	PD Allergen-specific IgE, kU/L to mite, egg white, and cow milk. Total fecal and total serum IgE kU/L	Allergic sensitization: Mean values of allergic sensitization to mite, egg white, and cow milk, and total fecal IgE level and total serum IgE level are reported in the original article. AD: NR	NR. But no probiotics or antibiotics therapy for at least 4 weeks before the sampling.	Metabolomic profile of rhinitis vs. asthma and compared to healthy controls.
Lee-Sarwar et al.[31] 2019 USA	Asthma 3 years old 203 (56)	Stool MS Untargeted	Nested case-control NR Black/White/Hispanic or other (%): 45/20/36	85 with asthma	276 without asthma	Parental report of a physician's diagnosis of asthma in the child's first 3 years of life	Allergic sensitization: in 49 with asthma (58%) and in 96 controls (35%). Eczema in 38 with asthma (45%) and in 62 controls (22%)	Recent use of ICS and/or systemic CS in the prior 3 months: 25 with asthma (29%) and 6 controls (2%)	Metabolomic profile of asthma vs. healthy
Carraro et al.[34] 2018 Italy	Wheeze Unborn, follow up at age 1 year 76 (53)	Amniotic fluid LC-MS and LC-MSE, Untargeted	Nested case-control The Netherlands NR	86 experienced at least 1 episode of wheezing in their first year	56 with no history of wheezing in their first year	Parental report at age 1 year: Parents were asked to record their children's respiratory symptoms (including wheezing) in a daily log.	Allergy: NR. But atopic parents: n=46 in the wheezing group and n=35 in the control group AD: NR	NR	Identify metabolites at birth associated with subsequent onset of wheezing during the first year of life.

Abbreviations: MS: mass spectrometry; LC: liquid chromatography; GC: gas chromatography; NMR: nuclear magnetic resonance; UPLC: Ultra performance liquid chromatography GINA: Global Initiative for Asthma; CS: corticosteroid; ICS; inhaled corticosteroid; NR: not reported; AD: atopic dermatitis; PD: physician-diagnosed; SPT; skin prick test; FA: food allergy; AR: allergic rhinitis; IgE: Immunoglobulin E; RSV: respiratory syncytial virus; HRV: human rhinovirus.

Table S2. PubMed database search.

A systematic search string was conducted by a combination of blocks of identified keywords and MeSH terms related to ‘atopy’ (asthma, allergy or atopic dermatitis), ‘metabolomics’ and ‘children’: *″Asthma″* OR *″Allergy″* OR *″Atopic dermatitis″* AND *″Metabolomics″* AND *″Children″*.

	**MeSH terms**	Keywords in “Title/Abstract”		
	Asthma	asthma		
″Asthma″	Bronchitis	bronchitis		
	RespiratorySounds/diagnosis	wheez*		
	Rhinitis, Allergic			
	Food hypersensitivity	“allergic rhinitis”	atopy	
″Allergy″		allergy	“allergic sensitization”	
		allergic	“food allergy”	
		"atopic dermatitis"		
″Atopic dermatitis″	Dermatitis, Atopic	"atopic eczema"		
		atopy		
		metabolome	metabolomic profil *	
		metabolite fingerprint *	metabolomics signatur *	
		metabolite phenotyp *	metabolomic signatur *	
	Metabolome	metabolite signatur *	metabolomics phenotyp *	metabolomic phenotyp *
″Metabolomics″	Metabolomics	metabolite profil *	metabolomic fingerprint *	metabolomics fingerprints
		metabolomics profil *	metabolomic *	
		metabolomic *	breathomic *	
		breathomic *	metabonomic *	
		metabonomic *		
		youth	teen	infant
	Infant	juvenile	baby	adolescent
″Children″	Adolescent	newborn	babies	preschool
	Child	young	kid*	children
		youngster	child	
